# Disparate Effects of Mesenchymal Stem Cells in Experimental Autoimmune Encephalomyelitis and Cuprizone-Induced Demyelination

**DOI:** 10.1371/journal.pone.0139008

**Published:** 2015-09-25

**Authors:** Justin D. Glenn, Matthew D. Smith, Leslie A. Kirby, Emily G. Baxi, Katharine A Whartenby

**Affiliations:** 1 Department of Neurology, Johns Hopkins University School of Medicine, Baltimore, Maryland, United States of America; 2 Department of Oncology, Johns Hopkins University School of Medicine, Baltimore, Maryland, United States of America; Wayne State University, UNITED STATES

## Abstract

Mesenchymal stem cells (MSCs) are pleiotropic cells with potential therapeutic benefits for a wide range of diseases. Because of their immunomodulatory properties they have been utilized to treat autoimmune diseases such as multiple sclerosis (MS), which is characterized by demyelination. The microenvironment surrounding MSCs is thought to affect their differentiation and phenotype, which could in turn affect the efficacy. We thus sought to dissect the potential for differential impact of MSCs on central nervous system (CNS) disease in T cell mediated and non-T cell mediated settings using the MOG_35–55_ experimental autoimmune encephalomyelitis (EAE) and cuprizone-mediated demyelination models, respectively. As the pathogeneses of MS and EAE are thought to be mediated by IFNγ-producing (T_H_1) and IL-17A-producing (T_H_17) effector CD4+ T cells, we investigated the effect of MSCs on the development of these two key pathogenic cell groups. Although MSCs suppressed the activation and effector function of T_H_17 cells, they did not affect T_H_1 activation, but enhanced T_H_1 effector function and ultimately produced no effect on EAE. In the non- T cell mediated cuprizone model of demyelination, MSC administration had a positive effect, with an overall increase in myelin abundance in the brain of MSC-treated mice compared to controls. These results highlight the potential variability of MSCs as a biologic therapeutic tool in the treatment of autoimmune disease and the need for further investigation into the multifaceted functions of MSCs in diverse microenvironments and the mechanisms behind the diversity.

## Introduction

Mesenchymal stem cells (MSCs) have potential therapeutic applications for a wide range of diseases as they offer many of the same benefits as embryonic stem cells without the logistical limitations. MSCs are a heterogeneous and multipotent population of stem cells with diverse functions that include protective and trophic effects such as inhibition of apoptosis and fibrosis, promotion of angiogenesis, progenitor cell maintenance, chemo-attraction, repair and both inhibition and enhancement of immunity, reviewed recently in [1].

MSCs have been shown to improve experimental models of several autoimmune diseases including Type 1 Diabetes, systemic lupus erythematosus, rheumatoid arthritis, and multiple sclerosis (MS) [1–5]. MS is a debilitating central nervous system (CNS) autoimmune disease that consists of CNS-directed inflammation, demyelination, and axonal degeneration. In one common murine model, experimental autoimmune encephalomyelitis (EAE), disease is initiated by auto-reactive T cells that are peripherally activated, migrate to and invade the CNS, become re-activated by resident antigen-presenting cells (APCs), and recruit additional peripheral pathogenic immune cells to contribute to the destruction of myelin and eventual neurodegeneration [2, 6]. MSCs were first shown to modulate CD4+ T cell-mediated MOG_35–55_ EAE by ameliorating the course of disease. This effect was associated with a reduction of demyelination, decreased T cell infiltration into the CNS, and induction of T-cell anergy [3–5, 7]. MSCs have been demonstrated to suppress important parameters of T cell activity including T cell activation, proliferation, production of pro-inflammatory cytokines such as IFNγ and IL-17A, and cytotoxicity [3, 4, 8–12]. Multiple MSC-derived products contribute to this immune-modulation including prostaglandin E_2_ (PGE_2_), nitric oxide from inducible nitric oxide synthase (iNOS), indoleamine-2,3-dioxygenase (IDO), truncated CCL-2 (tCCL-2), and membrane-bound adhesion molecules, and hepatocyte growth factor (HGF) [4, 5, 12–15].

Although MSCs have been shown to exert inhibitory immune-modulatory properties, additional studies have shown opposite effects. For example, MSCs were immunogenic in a model of graft-versus-host disease (GvHD) and induced a cytotoxic memory T cell response [16]. *In vitro* demonstrations of suppression have also not been recapitulated in some *in vivo* settings, as MSCs lacked significant effect on experimental autoimmune neuritis [17]. Furthermore, we have recently shown a differential effect of MSCs on different effector subsets of CD8+ T cells [18]. While MSCs suppressed Tc17 development, they enhanced IFNγ-producing CD8+ T cell function and exacerbated CD8+T cell-mediated MOG_37–50_ EAE. In our studies, MSCs enhanced early IL-2 production, which promoted Tc1 responses yet antagonized acquisition of the Tc17 program [18].

A growing literature in MS has focused on the roles of oligodendrocytes (OL) and neuro-protection in disease and therapy, independent of immune suppression [19]. A limitation of the standard EAE models is that it is difficult to separate the effects of therapies on immune suppression, which then leads to a decrease in immune-mediated demyelination, from direct toxic effects on neurons and/or OLs [2]. During demyelination, myelin-producing OLs undergo apoptosis and myelin loss [19, 20]. In response, oligodendrocyte progenitor cells (OPCs) proliferate and migrate to demyelinated areas to facilitate remyelination, but this remyelination process is typically incomplete or defective [19]. To assess the neuro-protective capacity of MSCs in a non-T cell mediated setting, models of chemically-induced demyelination, such as cuprizone and lysolecithin, have been employed. These models have the advantage of inducing demyelination via toxicity to OLs, without substantive involvement of the lymphocytic immune system and with predictable location and timing. Cuprizone is a copper chelator which results in reproducible demyelination of several brain regions including the corpus callosum and hippocampus [19, 21, 22]. Treated mice exhibit rapid and robust OL loss and demyelination followed by a period of remyelination. Although the effect of MSCs on inflammatory immune cells in neuro-degenerative disease is under investigation, little work has addressed the potential for MSCs to prevent demyelination *in vivo* by providing trophic support.

Despite conflicting reports of their effects on EAE, and a dearth of knowledge on how they impact non-T cell mediated demyelination, MSCs are currently being evaluated in human clinical trials for efficacy in MS. [3, 4, 18, 23, 24]. In order to more comprehensively address the effects of MSCs on neuro-autoimmune disease, we evaluated their action on neurological pathologies that were separated into models of either classical MOG-induced EAE or chemically induced demyelination. Our results indicate differential therapeutic efficacy within these two avenues and support the importance of dissecting the specific mechanisms that govern MSC responses in order to maximize their future therapeutic use.

## Materials and Methods

### Mice

Female C57BL/6 mice were purchased from the National Cancer Institute (NCI). NOD-SCID mice were purchased from Jackson Laboratories and bred in-house; females were used for all experiments.

### Ethics

All studies were approved by the Johns Hopkins University School of Medicine Animal Care and Use Committee. Mice were monitored daily by both laboratory personnel and veterinary staff and sacrificed as appropriate at signs of distress. For all experiments, animals were euthanized by isoflurane inhalation (Forane, Baxter Healthcare Corp., Deerfield, IL, www.baxter.com), followed by rapid cervical dislocation. For anesthesia in preparation for perfusions, animals were administered 5 mg/ml Nembutal Sodium solution (pentobarbital sodium injection, USP, Oak Pharmaceuticals, Lake Forest, IL, www.akorn.com protocols were followed to assure ethical treatment of animals, and veterinary care is provided for mice.

### *In Vitro* Culture and Characterization of MSCs

Murine MSCs were generated by conventional lab procedures: cells were harvested from the bone marrow of humerus and long bones, washed and cultured in Murine Mesencult medium (MMM) with stimulatory supplements (StemCell Technologies, Vancouver, Canada, www.stemcell.com) in 37°C/5% CO_2_ incubation. After 2 days of initial culture, non-adherent cells were removed and adherent cells (80–90% confluence) were passaged. Cells were passaged at 90% confluence and evaluated for cell surface phenotype beginning after the 10^th^ passage. Cells were previously characterized by FACS as CD9^+^CD44^+^CD73^+^MHC-1^+^Sca-1^+^ and CD11b^-^CD11c^-^CD34^-/lo^CD45^-^CD80^lo^CD86^-/lo^CD90^-^CD105^-^MHC-II^-^ [18]. MSCs were previously confirmed for their ability to functionally differentiate into adipocytes and osteoblasts [18].

For preparation of MSC-conditioned media (MSC-CM), MSCs were plated at 1x10^5^ cells/well in a 6-well plate in MMM alone or supplemented with 20 ng/ml recombinant mouse interferon-gamma (IFNγ) (eBioscience, San Diego, CA, www.ebioscience.com).

After 24–48 hr, media was discarded, cells were washed with PBS, and fresh MMM without additional factors was given to cells. Conditioned media from these MSCs was harvested after 24 hours and 0.22μm filtered.

### MOG_35–55_ EAE Induction, Behavioral Analysis and *Ex Vivo* T Cell Analysis

To induce EAE, 100 μg pure MOG_35–55_ in complete Freund’s adjuvant (CFA) (8mg/ml *M*.*tb*) (Thermo Scientific, Waltham, MA, www.thermoscientific.com and Difco Laboratories, Detroit, MI, www.bd.com, respectively) was injected in the abdomen subcutaneously on day 0. Pertussis toxin of 250 ng was administered intraperitoneally (i.p.) on days 0 and 2. For mice treated with MSCs, 5 x10^6^ MSCs in phosphate-buffered saline vehicle (PBS) were injected i.p. on days 3 and 8. Control mice received PBS vehicle. Mice were monitored daily by a blinded observer for behavioral EAE symptoms and were scored on a point system as previously reported [25]. Mice never progressed beyond EAE score 4. Suffering alleviated by adding mouse food pellets and hydration packs directly inside cages if mice were at least EAE score 2.5. For *ex vivo* T cell analysis, mice from 14 days after EAE immunization were killed, perfused with cold Hanks buffered saline solution, and whole brains were harvested. Brains were dissociated, filtered, and inflammatory infiltrates were isolated in Percoll gradients as previously reported [24]. Inflammatory cells were immediately restimulated with cell stimulation cocktail (as described) for 4 hours, stained, and analyzed by flow cytometry.

### *In Vitro* T-cell Polarization

Spleens were harvested from adult C57BL/6 mice and naïve CD4^+^ T cells were enriched using a Mouse CD4^+^ T Cell Enrichment Kit (StemCell Technologies), followed by selection of CD62L^+^CD4^+^ T cells using CD62L (L-selectin) microbeads (Milteny Biotech Inc., Bergisch Gladbach, Germany, www.miltenyibiotec.com). For activation, 5x10^5^ T cells were added to wells containing plate-bound anti-CD3 (5 μg/ml) and soluble anti-CD28 (2 μg/ml) in Iscove’s Modified Dulbecco’s Medium (IMDM)-based medium in six-well plates. For polarization, these activated cells were also administered skewing cytokines and cytokine-neutralizing antibodies. For generation of T_H_1 cells, cells were given IL-2 (5 ng/ml), IL-12 (10 ng/ml) (both from Peprotech, Rocky Hill, NJ, www.peprotech.com) and anti-IL-4 (20 μg/ml) (National Cancer Institute, NCI). For T_H_17 generation, cells were given IL-6 (20 ng/ml), transforming growth factor-β1 (TGF-β1, 5 ng/ml) (both from Peprotech), IL-23 (20 ng/ml), IL-1β (20 ng/ml) (both from R&D Systems, Minneapolis, MN, www.rndsystems.com), anti-IL-4 (20 μg/ml, NCI) and anti-IFNγ (20 μg/ml, eBioscience). For carboxyfluorescein succinimidyl ester (CFSE) labeling, 1x10^6^ cells per milliliter were labeled with 2.5 μg CFSE labeling dye (CellTrace CFSE Cell Proliferation Kit, Invitrogen, Carlsbad, CA, www.invitrogen.com). When used, MSCs were co-cultured at a ratio of MSC: T cell of 1:4.T cells were cultured for 72–120 hours before analysis.

### T Cell Intracellular Cytokine Analysis

T cells from polarization assays were re-stimulated with 2 μl/ml cells of Cell Stimulation Cocktail (eBioscience), which contains PMA/Ionomycin/Brefeldin-A/monensin, for 5 hours at 37°C. Cells were then stained with cell surface and intracellular antibodies using the Foxp3 staining buffer set (eBioscience). Antibodies used for cell analyses were conjugated to FITC, PE, PerCp, and APC, and are as follows: CD4-PerCp, IFNγ-FITC, (both from BD Biosciences, San Jose, CA, www.bdbiosciences.com), and IL-17A-APC (eBioscience). Cells were analyzed on a BD FacsCalibur.

### Cuprizone-mediated Demyelination

C57BL/6 or NOD-SCID mice were fed 0.2% w/w cuprizone (Bis(cyclohexanone)oxaldihydrazone, Sigma-Aldrich) in powdered irradiated global 18% protein rodent diet (Harlan, Indianapolis, IN, www.harlan.com) for 4 weeks (C57BL/6) or 8 weeks (NOD-SCID). The feed was changed 3 times per week. At designated time points, mice were administered i.p. injections of either MSCs (5x10^6^ MSCs/mouse/ time point in PBS) or PBS vehicle.

### Western Blot Analysis

Mice were anesthetized with isoflurane (Abbott Laboratories, Chicago, IL, www.abbott.com) and perfused through the left ventricle using chilled 1X Hank’s buffered saline solution [HBSS (Cellgro, Corning, NY, www.cellgro.com)]. Brains were removed and dissected under a Motic SMZ-168 microdissection microscope (Motic, Richmond, BC, Canada, www.motic.com). The corpus callosum from one hemisphere and hippocampus from the other were removed and immediately frozen on dry ice. The tissue was later thawed and homogenized on ice using a handheld homogenizer in RIPA buffer (Boston Bioproducts, Ashland, MA, www.bostonbioproducts.com) with protease and phosphatase inhibitors (both from Sigma-Aldrich, St. Louis, MO, www.sigmaaldrich.com). Protein concentration was determined by performing a Bicinchoninic Acid (BCA) assay with a Pierce BCA Protein Assay Kit (Thermo Scientific). Protein was separated by running 25μg (Corpus Callosum) or 40μg (Hippocampus) on NuPage 12% Bis-Tris Acrylamide Gels (Life Technologies, Carlsbad, CA, www.lifetechnologies.com) in NuPAGE MOPS SDS Running Buffer (Life Technologies). Separated protein was then transferred to Bio-Rad Midi nitrocellulose membranes (Bio-Rad, Hercules, CA, www.bio-rad.com) using a Bio-Rad Trans-Blot Turbo transfer system (Bio-Rad). Membranes were washed and blocked in 5% Non-Fat Milk in 1X Tris-Buffered Saline with .1% Tween-20 (TBS-T) for 1 hour at room temperature. Blocked membranes were then probed with primary antibody for myelin basic protein (MBP), SMI-99 (Covance, Princeton, NJ, www.covance.com) or for actin, AC-74 (mouse monoclonal anti-β-actin, Sigma-Aldrich) in blocking solution overnight at 4**°**C. Membranes were subsequently washed in TBS-T and then probed with secondary goat anti-mouse IRDye® 680RD or 800CW (Licor, Lincoln, NE, www.licor.com) in blocking solution for 1 hour at room temperature. Membranes were then washed and imaged using a Licor Odyssey (Licor). Quantitative measures were obtained using Image Studio 2.0 software (Licor).

### Black Gold Staining

Mice were anesthetized with sodium pentobarbital (Akorn Pharmaceuticals, Lake Forest, IL, www.akorn.com) and perfused through the left ventricle using chilled 1X HBSS followed by 4% w/v paraformaldehyde (Sigma-Aldrich). Brains were removed and post-fixed in 4% w/v paraformaldehyde overnight at 4°C. Afterwards, brains were moved to 30% w/v sucrose (Sigma-Aldrich) and kept at 4°C for approximately 48 hours. They were then frozen in isopentane (Sigma-Aldrich) and kept at -80°C until sectioning. 20μm sections were obtained on a cryostat and transferred to superfrost plus microscope slides (Fisher Scientific, Waltham, MA, www.fishersci.com). Sections were stained using Black Gold II Myelin Staining Kit (EMD Millipore, Billerica, MA, www.emdmillipore.com) according to manufacturer’s instructions and imaged on an Olympus BX41 microscope (Olympus, Waltham, MA, www.olympus-lifescience.com).

### Rat OPC Isolation and *Ex Vivo* Culture

Rat oligodendrocyte precursor cells were cultured by dissecting cortices from neonatal P6 Sprague Dawley rats (Charles River) in chilled 1X HBSS. Cortices were diced and dissociated using the MACS neural tissue dissociation kit (Miltenyi Biotec). A2B5 positive cells were selected using MACS A2B5 Microbeads (Miltenyi Biotec) according to manufacturer’s instructions. A2B5 positive cells were cultured on Poly-L-Lysine pretreated culture dishes in Sato media [modified from [26]] (Dulbecco’s Modified Eagle Medium (Invitrogen) containing 2 mM glutamine (Invitrogen), 100 U/ml penicillin, 100 μg/mL streptomycin (Life Technologies), 1 mM sodium pyruvate (Invitrogen), 5 μg/ml insulin (Sigma-Aldrich), 5 ug/ml N-Acetyl-L-cysteine (Sigma-Aldrich), trace elements B (Cellgro), 50 ng/ml hydrocortisone (Sigma-Aldrich), 10 ng/ml d-Biotin (Sigma Aldrich), B-27 (Invitrogen), 100 μg/ml Bovine Serum Albumin (Sigma-Aldrich), 100 μg/ml apo-Transferrin (Sigma-Aldrich), 16 μg/ml putrescine (Sigma-Aldrich), 60 ng/ml progesterone (Sigma-Aldrich), and 40 ng/ml sodium selenite (Sigma-Aldrich)) containing 20 ng/ml recombinant human PDGF-AA (Peptrotech) to promote proliferation. The media was refreshed after approximately 2 days in culture. Three-to-four days after plating, media was discarded, cells were washed with 1X PBS, and given a 1:1 ratio of Sato medium: MMM or 1:1 ratio of Sato medium and either un-stimulated MSC-CM or CM from IFNγ-stimulated-MSC (from which the initial IFNγ had been washed out).

### *In vitro* OPC Flow Cytometric Analysis

After 7–9 days of OPC culture in MSC-CM, OPCs were harvested from plates by micropipette and stained for flow cytometry. For MBP staining, OPCs were stained with the myelin basic protein monoclonal antibody SMI99 (Covance), followed by AlexaFluor 488-conjugated, F(ab’) 2 goat anti-mouse IgG (H+L) secondary antibody staining (Life Technologies). For evaluation of apoptosis, cells were stained with Annexin V-APC using the Annexin V Apoptosis Detection kit (eBioscience). Cells were analyzed on a BD FacsCalibur.

### *Ex vivo* Oligodendrocyte Flow Cytometry

Female C57BL/6 mice were cuprizone treated and administered MSCs or PBS as previously described. After 14 days of treatment, mice were anesthetized with isoflurane and perfused through the left ventricle using chilled 1X Hank’s buffered saline solution. Corpora callosa were dissected from brains under a Motic SMZ-168 microdissection microscope and were then manually minced and processed for single cell suspension as described [27] for flow cytometry. For oligodendrocyte identification, cells were stained with anti-Galactocerebroside, clone mGalC, Alexa Fluor488 Conjugate (EMD Millipore). For evaluation of death, cells were stained with Annexin V-APC (BD Biosciences) and 7-AAD (BD Pharmingen) as suggested by manufacturers. Cells were analyzed on a BD FacsCalibur.

### Statistics

All statistics were conducted using GraphPad Prism (GraphPad, San Diego, CA).

## Results

### Mesenchymal stem cells do not impact the course of CD4+ T cell-mediated EAE

**Fig 1:**
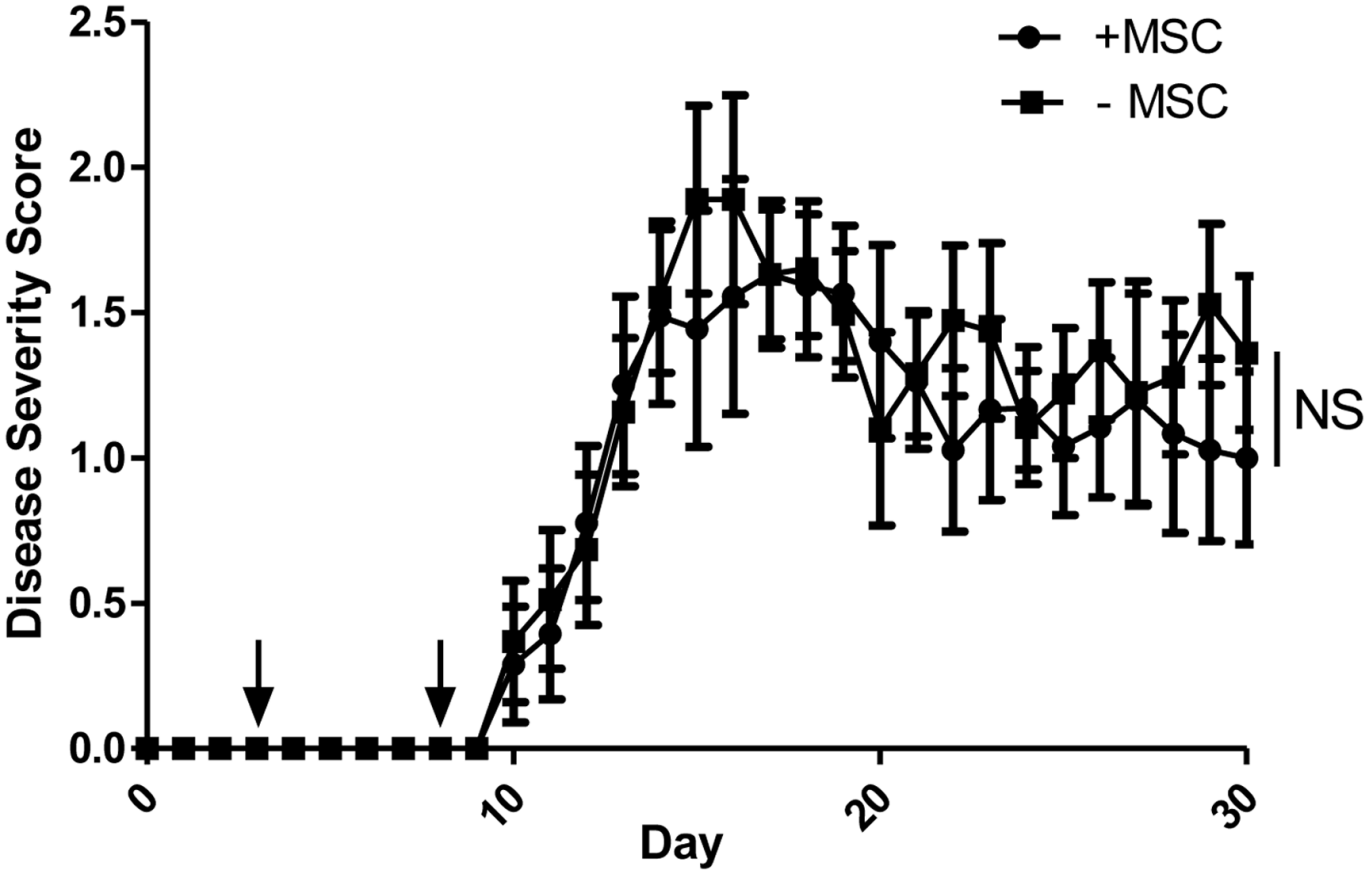
MSCs do not affect the MOG_35–55_ EAE disease course. C57BL/6 mice were immunized with MOG_35–55_ in complete Freund’s adjuvant. At days 3 and 8 post-induction (black arrows), mice were administered either murine MSCs or phosphate-buffered saline vehicle. Mice were scored daily in a blinded fashion. Data shown are a combination of two independent experiments. Statistical analysis was done with the Mann-Whitney *U*-test.

We assessed the potential of MSCs to improve the disease course in the classical MOG_35–55_, CD4+ T cell-initiated EAE. Mice were immunized and then administered two doses of MSCs during the priming phase of disease. With this regimen, the MSCs had no impact on either the duration or severity of EAE disease (Fig 1).

### Mesenchymal stem cells differentially affect the development of effector CD4+ T cell subsets

We next conducted *in vitro* studies to investigate the effects of MSCs on specific T cell subsets involved in EAE. Spleen-derived, naïve CD62L+ CD4+ T cells were activated with α-CD3/α-CD28 and either kept activated but un-polarized or polarized towards T_H_1 cells with the cytokines IL-2 and IL-12. We also polarized T cells towards two different EAE-associated T_H_17 cell populations with the cytokines IL-6 and TGF-β1 ± the cytokines IL-23 and IL-1β [6, 28, 29]. MSCs were added to the polarizing cells and T cells were analyzed for proliferation and canonical cytokine production 3 days post-activation.

**Fig 2:**
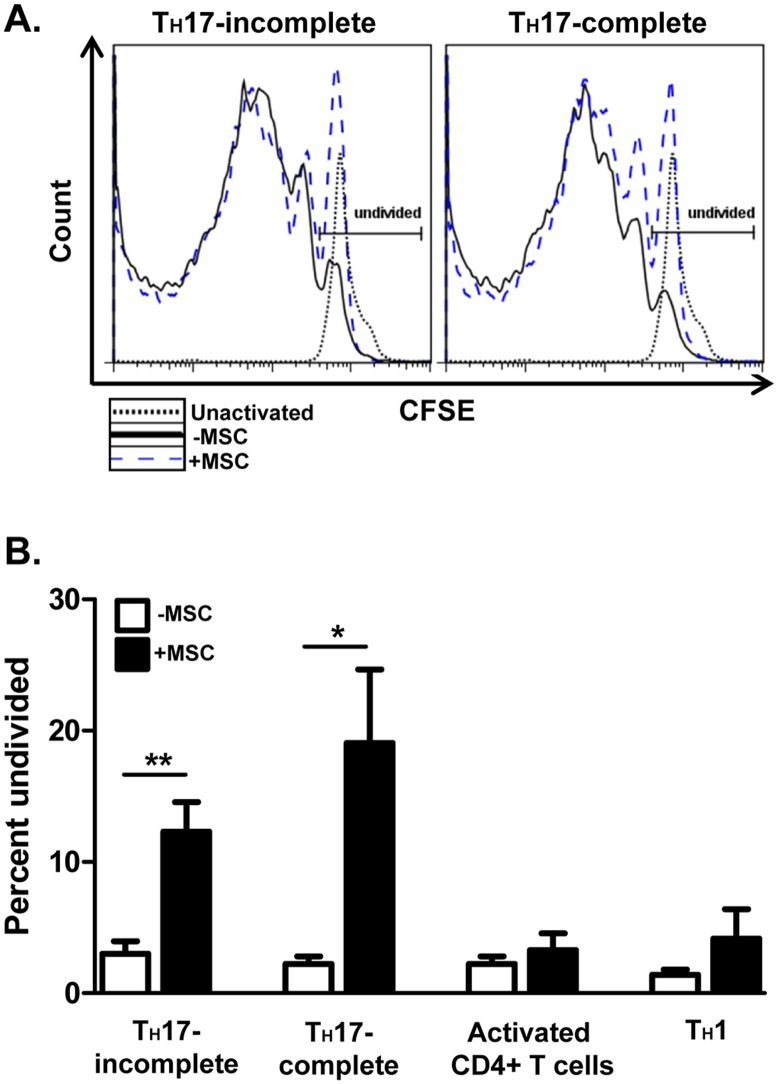
MSCs differentially affect the proliferation of effector CD4^+^ T cells *in vitro*. Naïve CD62L^+^CD4^+^ T cells were cultured in the absence or presence of MSCs at a T cell: MSC ratio of 4:1. CD4+ T cells were activated with plate-bound α-CD3 and soluble α-CD28 ± polarizing cytokines and neutralizing antibodies. After 72 hours, T cells were harvested, re-stimulated with cell stimulation cocktail for 5 hours, stained, and analyzed by flow cytometry. Shown are representative histograms of CFSE dilution, gated on CD4^+^ T cells, of undivided incomplete T_H_17 cells (without IL-23/IL-1β) and complete T_H_17 cells (with IL-23/IL-1β) **(A)**, and bar graphs of the undivided incomplete and complete T_H_17 populations, activated CD4^+^ T cells, and T_H_1 cells **(B)**, in the absence and presence of MSCs. Compiled bar graph data are from three separate experiments (n = 5/treatment group). Significance was measured by Student’s *t*-test, with *p<0.05 and **p<0.01.

MSCs exerted differential effects on CD4+ T cells, depending on the T cell effector subset. MSCs dramatically inhibited T_H_17 cell proliferation (Fig 2A). Interestingly, MSCs still significantly suppressed T_H_17 proliferation when the T_H_17-enhancing cytokines IL-23 and IL-1β were added. In contrast, activated and T_H_1 cells proliferated to a similar extent regardless of MSC presence, though MSCs tended to increase the percentage of undivided cells (Fig 2B).

**Fig 3:**
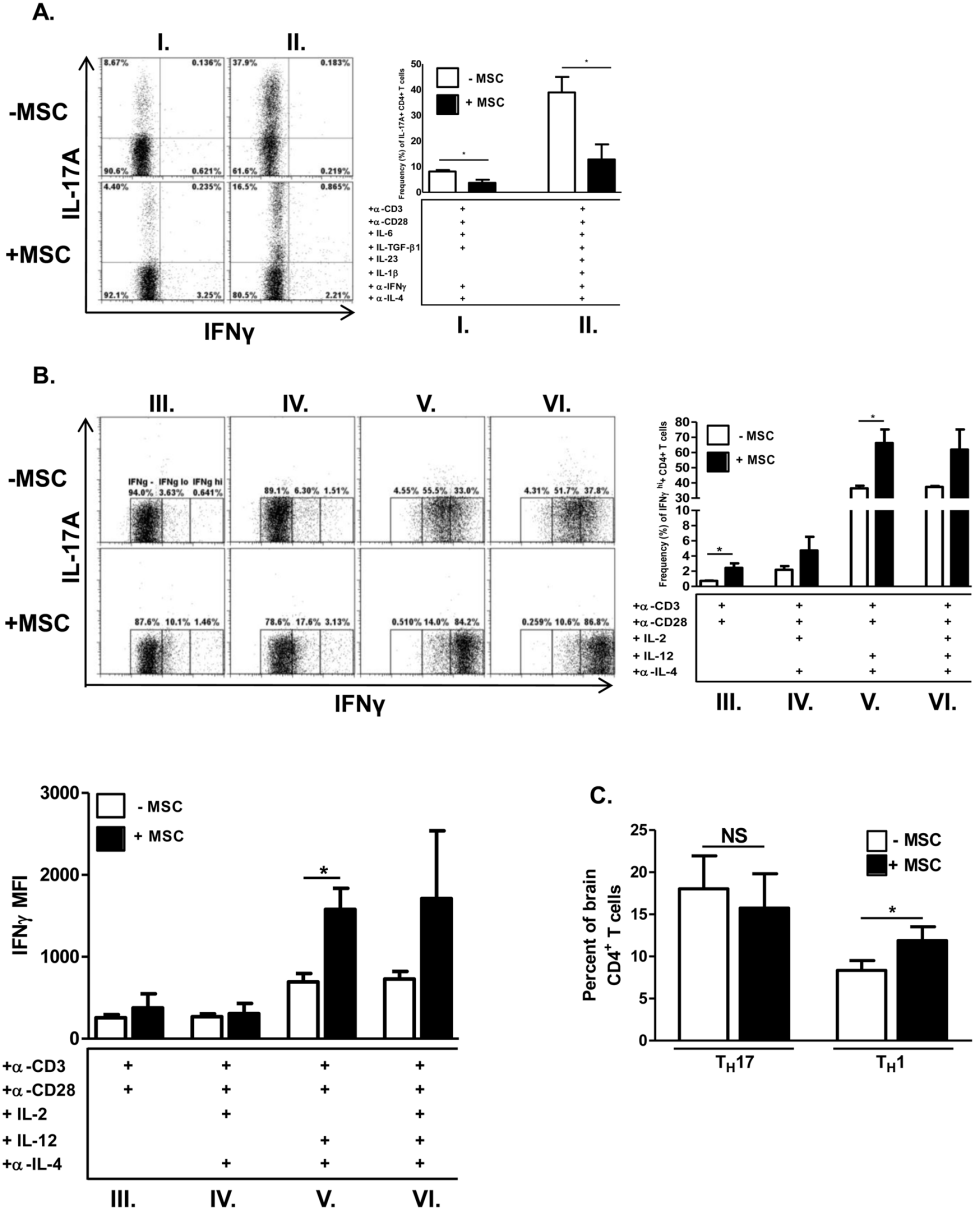
MSCs differentially affect cytokine production in effector CD4^+^ T cells *in vitro* and *in vivo*. For *in vitro* experiments, naïve CD62L^+^CD4^+^ T cells were cultured in the absence or presence of MSCs at a T cell: MSC ratio of 4:1. CD4+ T cells were activated with plate-bound α-CD3 and soluble α-CD28 ± polarizing cytokines and neutralizing antibodies. After 72 hours, T cells were harvested, re-stimulated with cell stimulation cocktail for 5 hours, stained, and analyzed by flow cytometry. Shown are representative flow cytometric plots, gated on CD4^+^ T cells, of T_H_17 cells **(A)** and activated CD4^+^ T cells and T_H_1 cells **(B)**, in the absence and presence of MSCs. Compiled bar graph data are from three separate experiments. Roman numerals refer to specific polarization conditions as shown. For (B), IFNγ mean fluorescence intensity (MFI) values are shown and were calculated from CD4-subgated IFNγ^+^ cells. Compiled bar graph data are from two independent experiments. For *in vivo* experiments **(C)**, female C57BL/6 mice were immunized with MOG_35–55_ and complete Freund’s adjuvant for EAE induction as previously described (n = 5/treatment group). On days 3 and 8 post-immunization, mice received either murine MSCs or phosphate-buffered saline vehicle as previously described. Brains were harvested 14 days post-immunization and processed for analysis of infiltrating lymphocytes, which were then analyzed by flow cytometry for frequencies of IFNγ^+^ T_H_1 cells and IL-17A^+^ T_H_17 cells. Shown are compiled bar graph data from two separate experiments. Significance was measured by Student’s *t*-test, with *p<0.05.

MSCs strongly suppressed differentiation into T_H_17 cells, even with the exogenous addition of the IL-17-lineage stabilizing and expansion cytokines IL-23 and IL-1β (Fig 3A). Activated, un-polarized CD4+ T cells do not produce significant quantities of IL-17A but do produce low levels of IFNγ. During MSC co-culture, these cells increased IFNγ production, albeit modestly (Fig 3B). The pro-inflammatory cytokine IL-12 potently induces IFNγ expression and drives T_H_1 development. Even after IL-12 administration, MSCs significantly enhanced the frequency of IFNγ-producing T_H_1 cells and level of IFNγ production.

Comparable to our *in vitro* studies, we found that mice undergoing EAE exhibited a significantly higher frequency of T_H_1 cells in brains with MSC treatment, though the T_H_17 cell frequency was unaffected (Fig 3C).

### MSCs ameliorate cuprizone-mediated demyelination in the corpus callosum

**Fig 4:**
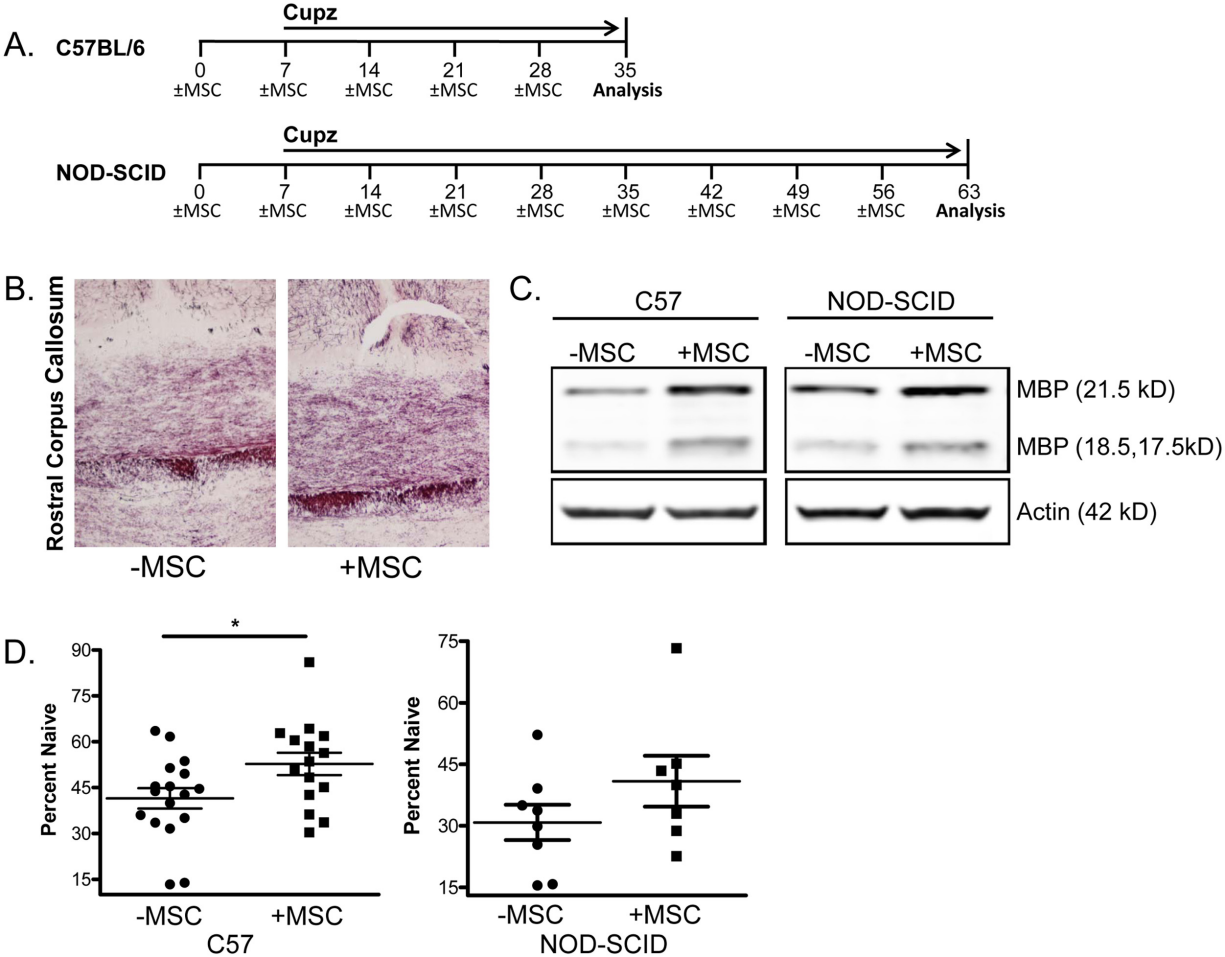
MSCs modestly suppress demyelination in cuprizone-treated mice. **(A)** C57BL/6 (C57) or NOD-SCID (N-S) mice were fed cuprizone (Cupz, 0.2 w/w)-containing rodent diet continuously for either 4 (C57) or 8 (N-S) weeks, concomitant with weekly i.p. injections of either MSCs or PBS vehicle, which were typically initiated one week prior to cuprizone feed. Mice were then euthanized for downstream brain myelination analyses. MSC administration resulted in higher quantities of myelin, as evidenced by Black Gold staining (C57 rostral corpus callosum (CC) **shown in B)** and by Western blot probing of MBP large (21.5 kD) and small (18.5 and 17.5 kD) isoforms in C57 CC and N-S hippocampus(Hp) **(C)**. Quantitative myelin measurements from either C57 or N-S-derived combined large and small MBP isoforms were generated from Western blot densitometries **(D)**. Each graph shows the combined results from two individual experiments (n = 15–17 C57and 7–8, (N-S). Significance was measured by Student’s *t*-test, with *p<0.05.

Within the EAE model, much of the pathology results secondary to the immune response, and thus we next sought to assess the specific effects of MSCs on the abundance of myelin, independently of its effects on the lymphocytic compartment of the immune system. To this end, we tested the impact of MSC therapy on cuprizone-mediated demyelination, which causes a less-inflammatory, chemically-induced demyelination of the corpus callosum and hippocampus. C57BL/6 mice were kept on cuprizone feed continuously and MSCs were injected on a weekly basis. After four weeks, mice were euthanized and their brains dissected and analyzed for myelin content via two different strategies (Fig 4A–4C). We analyzed focal effects by using the myelin stain Black Gold and quantified total myelin content by Western blot analysis of the affected regions of the brain. To eliminate the possibility of B and T cell-mediated immune effects on demyelination, we extended our cuprizone studies to NOD-SCID mice. In our experiments, while C57BL/6 mice exhibited significant demyelination after 4 weeks on cuprizone, there was a delayed effect in NOD-SCID mice, which did not reveal substantial demyelination until after 8 weeks. In addition, while C57BL/6 mice exhibited demyelination in both corpus callosum and hippocampus, the demyelination in NOD-SCID mice was primarily localized to the hippocampus.

As shown by the myelin stain Black Gold, mice that received MSCs had higher quantities of myelin (Fig 4B). Quantitatively, MSC administration produced a significant albeit modest effect on myelin abundance, as shown from the Western analysis (Fig 4C and 4D). A similar trend was observed in NOD-SCID mice, although the effect fell slightly short of statistical significance (Fig 4D).

### MSCs decrease oligodendrocyte death

**Fig 5:**
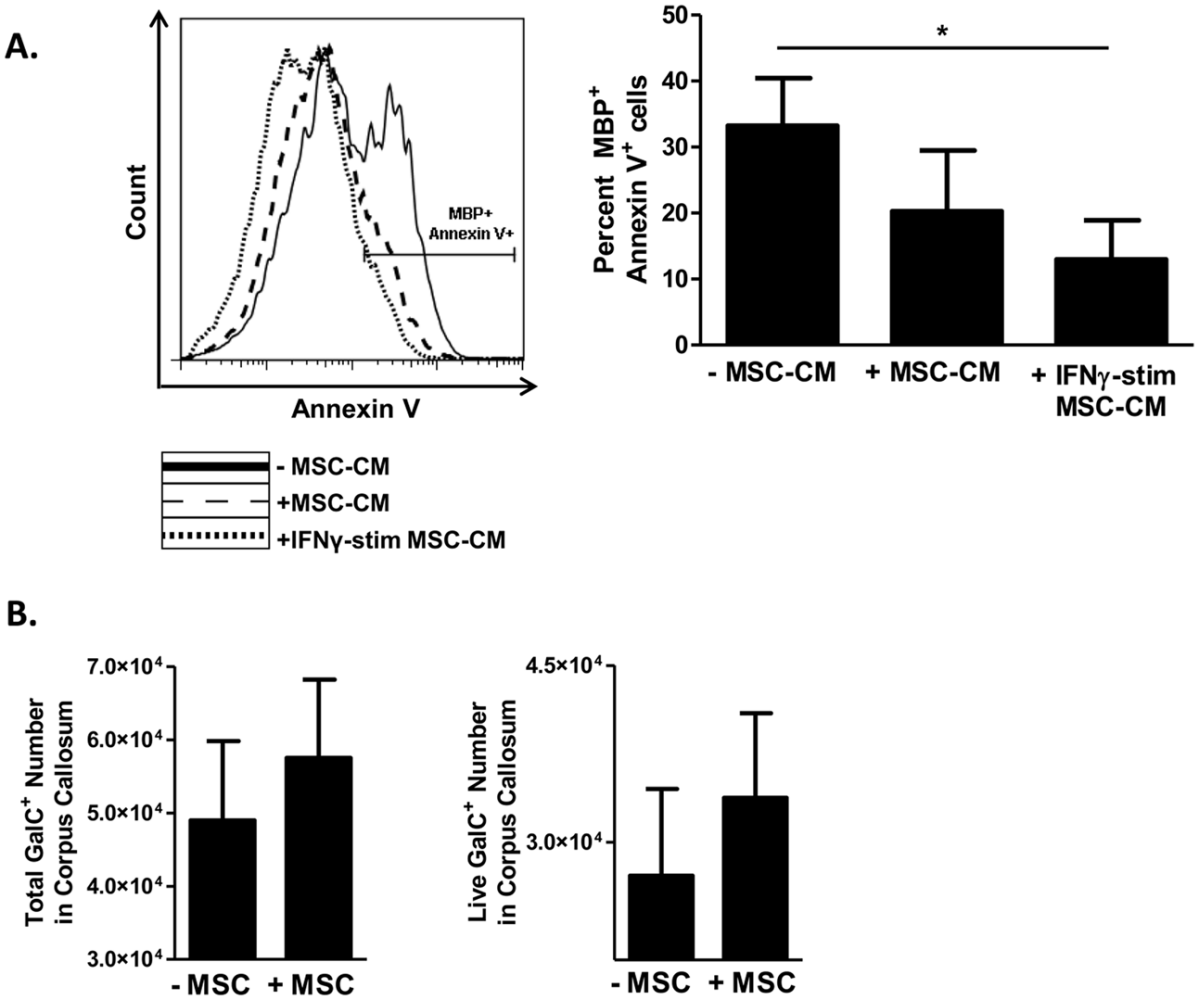
MSCs decrease oligodendrocyte death. **(A)** Oligodendrocyte progenitor cells (OPCs) were isolated from neonatal rat brains and cultured in PDGF-supplemented Sato medium. For preparation of MSC-conditioned media (MSC-CM), MSCs were plated in Mouse Mesencult medium (MMM) alone or supplemented with interferon-gamma (IFNγ). After 24–48 hr, media was discarded, MSCs were washed with PBS, and subsequently received fresh MMM without additional factors. Conditioned media from these MSCs were harvested after 24 hours and filtered. After 3–4 days of culture in PDGF-supplemented Sato medium, OPCs then received 1:1 ratio of Sato medium: MMM (-MSC-CM, conditioned-medium), 1:1 of Sato: un-stimulated MSC-CM (+MSC-CM), or 1:1 of Sato: IFNγ-stimulated MSC-CM, for a period of 7–9 days. Cells were then harvested and MBP/Annexin V-stained for downstream flow cytometric analysis. Cells were gated on MBP+ cells for Annexin V analysis. Shown are a representative histogram and a bar graph consisting of data from three independent experiments. **(B)** To evaluate the effect of MSC action on oligodendrocyte death *in vivo*, female C57BL/6 mice were cuprizone-fed and treated with MSCs or phosphate-buffered saline vehicle as previously described (n = 5/treatment group). After 14 days of treatment, corpora callosa were harvested from brains and manually minced. Single cell suspensions were prepared, stained for the oligodendrocyte marker galactocerebroside (GalC) and for Annexin V and 7-AAD to evaluate cell survival (Annexin V^neg^/7-AAD^neg^ cells). Cells were analyzed by flow cytometry. Shown are compiled bar graph data from two independent experiments. Significance was measured by Student’s *t*-test, with *p<0.05.

We next assessed whether MSCs might have direct effects on myelin-producing cells *in vitro* that might at least partially contribute to the observed effect of MSC treatment. Murine Mesencult Media (MMM) or IFNγ-containing MMM (for MSC immune stimulation) was given to MSCs for 24–48hr. These media were then removed from MSCs and these cells were washed with PBS before the addition of fresh MMM, which was then harvested after 24hr, filtered, and used as MSC-CM for OPC culture. OPCs were isolated, expanded for 3 days, and then exposed to conditioned media from un-stimulated MSCs or MSCs stimulated with the pro-inflammatory cytokine interferon-gamma (IFNγ), as MSC function has been shown to be influenced by inflammatory environments [14]. Effects of MSC-CM on differentiation-induced cell death of MBP-expressing OLs were quantified by FACS analysis of Annexin positivity on MBP+ OLs. Conditioned medium harvested from un-stimulated MSCs inhibited apoptosis of MBP+ OLs (Fig 5A). This inhibitory effect was enhanced when conditioned medium was harvested from MSCs that had been pre-treated with the inflammatory cytokine IFNγ. Thus MSC-mediated inhibition of OL apoptosis may contribute to the increased amount of myelin measured in cuprizone-mediated demyelination.

To address the *in vivo* relevance of MSC effects on oligodendrocyte survival, we administered MSCs to mice undergoing cuprizone-mediated demyelination and evaluated the survival of galactocerebroside (GalC)^+^ oligodendrocytes from the corpus callosum. MSC treatment resulted in a modest positive effect on both the total number of oligodendrocytes and on cell survival (Fig 5B).

## Discussion

Multiple pre-clinical therapies have been proposed for the amelioration of autoimmune disease, including bi-specific antibody and antibody multimer treatment, oral tolerance induction, and peptide immunotherapy [30–42]. These approaches have the advantage of adding specificity to the target tissue to be protected from immune attack, but may be limited in practicality by many variables, including inability to reach the target issue for protection, non-cell specific expression of target tissue antigens, location of antigen expression, and the potential inability of toleragenic cells to suppress activated, memory auto-antigenic T cells. In addition, in the context of neuro-autoimmune disease such as MS, demyelination may occur independent of inflammation, in which case these therapies would be of little use [19]. Hence there is a need for therapies that suppress inflammation and promote regeneration of damaged tissue as a result of autoimmune disease.

MSCs possess a number of distinctive characteristics that make them very appealing as a therapeutic modality. Their plasticity and diverse functions allow them to contribute to the repair and healing of multiple types of diseases [2–4, 8]. This varied nature, however, has a double edge to it, in that MSCs may have the potential to acquire undesired properties which may be influenced by the environment in which either they are produced or to which they migrate. Given their differential effects, the present studies sought to evaluate their impact on two distinct elements that characterize the disease process of MS, namely the autoimmune response and demyelination.

Pre-clinical investigations of the impact of MSCs on the widely used immune based rodent MS model, EAE, have yielded mixed results [3, 5, 18, 23, 24]. There are a number of important differences in the model systems tested, which may at least partially account for some of the disparate outcomes. The types of immune responses along with the state of the immune system at the time of delivery (e.g., in an activated or suppressed state) and the subtype of predominant T cell response could all contribute to differences, and further identifying the mechanisms of action of these cells will be critical to greater understanding.

To more extensively determine the effects of MSCs on the inflammatory and non-inflammatory events of neuro-degenerative disease, we evaluated the impact of MSCs on both the immune-generated model, MOG_35–55_ EAE, which relies on the induction of an autoreactive T cell response and on the cuprizone model of myelin destruction, which produces a chemically-induced demyelination. Surprisingly, we found that MSCs differentially affected each disease process, producing no detectable impact on disease progression in immune-mediated EAE, but exerting a positive effect on myelin abundance in cuprizone-mediated demyelination.

MOG_35–55_ EAE is principally mediated by pro-inflammatory, myelin-reactive IFNγ-producing T_H_1 and IL-17A-producing T_H_17 cells [20]. These cells migrate from their sites of activation, break down the blood-brain barrier, invade the CNS and initiate the process of myelin destruction and axonal degradation [19, 20]. In some reports, MSCs were shown to ameliorate EAE, presumably by decreasing T cell activation, proliferation, cytokine production, and induction of T cell anergy [5, 7–12]. These effects have been attributed to diverse MSC-derived products and effects, including truncated CCL-2 (tCCL-2), hepatocyte growth factor (HGF), and induction of regulatory CD4+ T cells (Tregs) [5,7,8,9,10,11,12]. However, other contrasting reports have shown MSC-induced exacerbation of EAE and increased pathogenic T cell brain infiltration [23, 43, 44]. These findings were demonstrated to be dependent on MSC dosage and involvement of CD8+ T cells in pathogenesis.

In previous studies, we reported that MSCs exacerbated MOG_37–50_ EAE, which is predominantly mediated by pro-inflammatory Tc17 cells and Tc1 cells [18, 45]. Interestingly, we found that MSCs differentially affected the development of effector CD8+ T cells depending on the specific subtype. While MSCs potently prevented Tc17 development, they enhanced IFNγ production and cytotoxicity of activated, non-polarized CD8+ T cells. These cellular effects relied on the early T cell production of IL-2, which is enhanced by MSC co-culture. Considering the differential effect of MSCs on effector CD8+ T cell development, we evaluated the effect of MSCs on discrete effector subsets of CD4+ T cells. Consistent with our previous studies on CD8+ T cells, MSCs increased IFNγ production in activated, non-polarized CD4+ T cells, with a more noticeable increase in the presence of IL-12, while reducing the frequency of T_H_17 cells, as has been extensively observed in other studies [11–13]. MSCs also increased the frequency of T_H_1 cells in the brains of MOG_35–55_ EAE mice. These effects on the generation of CD4+ T cell effector subsets known to cause disease may provide one possible explanation of the failure of the MSCs to modulate MOG_35–55_ EAE pathogenesis in this setting and have relevance to MS in which it has been postulated that heterogeneity of the disease may be partly determined by relative propensity for T_H_1 vs T_H_17 immune deviation [43].

Ultimately, demyelination and subsequent axonal pathology lead to disability in MS [2, 19, 44], and many current therapies are targeting neuroprotection and remyelination. To assess the protective effects of MSCs, while minimizing confounding immune effects, we employed the cuprizone model of demyelination and OL toxicity [26, 28, 29]. While the EAE model has many appealing features that make it relevant to the study of MS, one limitation is the intertwined nature of the immune response and demyelination. As demyelination is a direct result of the immune response, therapeutic interventions that suppress the immune response generally suppress the demyelination. However, progressive demyelination, with subsequent axon injury and loss, may occur somewhat independently of inflammation or persist after immune resolution [46, 47]. The cuprizone model allows an evaluation of the potential for MSCs to directly impact de/re-myelination in a less immune-dependent setting, independent of B and T cells. Our results revealed that mice fed cuprizone and treated with MSCs had higher quantities of myelin than did cuprizone-fed untreated mice. This result is consistent with two previous reports demonstrating positive effects of MSCs on demyelination. One group reported that grafting of MSCs led to an increase in OPC migration to the lesion, remyelination, and axon conduction velocity. This group hypothesized that the secretion of trophic factors plays a significant role in the beneficial effect [46]. A second report utilized adipose-derived MSCs, which were injected i.v., and also found a beneficial effect of the MSCs on the quantity of myelin [48]. One contrasting study showed no significant effect of MSCs on demyelination [49], although there were numerous differences in experimental design, including a substantially shorter time frame after administration of MSCs (3.5–7 d) prior to analysis, which may have contributed to these differences.

The isolation of individual variables in multifactorial models is complex, and while cuprizone is generally considered to be non-immune mediated, some studies have suggested that there is a contributing immune element [50, 51]. As many of the immune effects of MSCs have been ascribed to their actions on T cells, we assessed whether MSCs would produce a similar effect in immune-deficient mice. As the NOD-SCID mice lack B and T cells, this model provides a useful system for determining whether the observed effects of MSCs on myelin abundance might be due to secondary effects on lymphocytes. The results of our studies showed a similar trend towards a beneficial effect in the immunocompromised mice, further supporting the hypothesis of a direct role of MSCs on OPCs and/or OLs.

One possible explanation for our finding of beneficial effects on myelin abundance is that MSCs directly impact the survival of OPCs and/or OLs during apoptosis, possibly resulting from their trophic effects. We thus evaluated a culture system in which conditioned medium from MSCs was added to differentiating OPCs. Our results revealed that factors secreted by MSCs led to a significant survival advantage for the MBP+ OLs. In addition, MSCs exerted a positive effect on oligodendrocyte survival *in vivo*, as demonstrated in our studies of MSC administration to mice undergoing cuprizone-mediated demyelination. These results are consistent with previous studies in which MSC production of factors such as HGF positively impacted the generation of OPCs and neurons from cultured neurospheres [5]. Additional studies also showed that MSCs produced anti-apoptotic and growth factors that together exerted a protective effect on neural axons [52, 53]. Thus MSCs may simultaneously modulate different aspects of pathogenic processes, which is of particular significance in MS/EAE, since there can be both an immune-mediated and a direct demyelinating component [2, 44].

Taken together, these results indicate the potential for MSCs to act on the CNS directly and produce a beneficial effect on myelination in a toxic environment.

While MSCs clearly have potent therapeutic capabilities, a greater understanding of their plasticity within different settings will be necessary to optimize their clinical use. Mechanistic analyses of their various functions may help to reveal the specific nature of the separate functions that lead to these outcomes. Ultimately, identifying the diverse and individual capabilities of MSCs will help to harness their potential.

## Supporting Information

S1 Checklist ARRIVE NC3Rs Guidline Checklist. (PDF)
